# Educating the whole person: Broad extracurricular involvement and prevalence of purpose and thriving among college students in China

**DOI:** 10.3389/fpsyg.2022.1001766

**Published:** 2022-11-14

**Authors:** Wenling Zhang, Jenni Menon Mariano, Mo Zhu, Fei Jiang

**Affiliations:** ^1^Center for Ideological and Political Education, Northeast Normal University, ChangChun, China; ^2^Department of Educational and Psychological Studies, University of South Florida, Tampa, FL, United States

**Keywords:** purpose in life, extracurricular activities, college students, China, person-centered analysis

## Abstract

College students in China can choose from a wide variety of university organized extra-curricular activities (EAs), which are intended to enrich and deepen the learning experience, and reflect an educational policy goal to cultivate the whole and well-rounded person. These activities are also consistent with another policy goal, which is to foster optimal student outcomes like life purpose development. This study examined associations between EA involvement and life purpose and associations between EA involvement and thriving attributes of resourcefulness/resilience and life satisfaction among 332 undergraduate students enrolled at 20 universities across China. Measures of EA involvement, purpose resourcefulness/resilience, and life satisfaction were adapted to the Chinese culture, language, and student population. Four clusters of students emerged, representing varying degrees of well-roundedness according to their EA involvement. Patterns of associations examined between and within clusters supported the general hypothesis that more frequent and well-rounded EA involvement is positively associated with purpose and life-satisfaction, but not with resilience/resourcefulness. The significance of the findings for future research and practice are discussed.

## Introduction

Many educators worry about whether, and how, their education systems are preparing students to face the challenges of contemporary society, now and into the future. In China, national policies in higher education have shifted in recent years, in part to place special attention on meeting the needs of the widening diversity of students entering universities as the nation seeks to improve the quality of the college learning experience. These policies underscore the goal of all-round and whole-student growth, including nurturing students’ moral and personality development, “promoting the healthy growth of students as the starting point and end point” of schoolwork, encouraging students’ sense of initiative, and by promoting a comprehensive approach to learning through the “organic integration” of ethical, intellectual, physical, and aesthetic aspects of education (Ministry of Education of the People’s Republic of China, 2010; Xinhua News Agency, 2020). As such, while the curriculum differs across institutions depending on their particular missions and cultures, Chinese universities are all offering deeper and broader learning experiences than before (Wong, 2020; Lan and Zhang, 2021; i.e., see Xinhua News Agency, 2020).

This approach to student cultivation is also consistent with views in developmental science, which point to the college context and the periods of adolescence and emerging adulthood as special occasions for individuals to develop the foundations of a life of purpose (Malin, 2022, p: 1). Structured opportunities that take place outside of regular classes may be associated with students’ purpose by assisting young people to reflect “on their social responsibilities” their “value system,” and “the roles that they might play in contributing to society” (Malin, 2022, p: 1), which are all foundational to purpose development. Also, because involvement is voluntary, these opportunities represent students’ choice and initiative even beyond compulsory curricular experiences that aim to do the same things; and this choice in turn reflects the agentic and self-directed nature of purpose (Reilly and Mariano, 2021).

An important question is whether involvement in voluntary activities as organized in Chinese universities is in fact associated with students’ optimal outcomes – particularly with purpose. It is surprising that more attention has not been given to studying purpose within the college context since finding a purpose impacts young people’s well-being and is in line with the aims of higher education in many places (Pfund et al., 2020, p: 98). In fact, purpose is associated with well-being outcomes in emerging adulthood like life satisfaction and hope, and can help predict student success during university as it is associated with student self-efficacy, persistence in college, and mental health (Pfund et al., 2020). In considering purpose’s benefits, the comprehensive approach to knowledge integration proposed by higher education policy in China is of particular interest here. There is an assumption that exposure to a plethora of knowledge systems fosters well-roundedness, which in turn will lead to positive student outcomes. But a caveat that could disrupt this idea is that students who enter universities are increasingly diverse: not just in their demographic features like gender, or cultural and socio-economic background which are often measured as proxies for diversity, but also in terms of students’ personal interests and purpose development status. Thus, universities will be well served to understand the full diversity of students’ extracurricular choices, as well as their purpose status, so as to inform the design of programs that will truly reach all students (Malin, 2022).

In the following pages, we first discuss definitions of purpose, including Chinese perspectives. We then describe research on purpose and extracurricular activities in college. In doing so, we give special attention to the application of these ideas to students’ experiences in contemporary higher education in China. Next, we describe the current study, which investigated the relationship between organized extracurricular activities (EAs) and purpose features and between EAs and two thriving features (resourcefulness/resilience and life satisfaction) among Chinese college students. As shown further on, EAs in Chinese universities factor into a variety of distinct types, reflecting the multi-knowledge systems that higher education policy seeks to integrate. Thus, this study also allows us to examine whether well-rounded involvement in a variety of EAs is in fact associated with positive outcomes of purpose and thriving.

### Purpose and extracurricular activities in Chinese perspective

McKnight and Kashdan (2009) posit that purpose is “… a central, self-organizing life aim that organizes and stimulates goals, manages behaviors, and provides a sense of meaning” (p: 242). Thus, having a purpose can act like a compass for students as they formulate their life goals and plan pathways for their future (Malin, 2022). Purpose may also include an “other-oriented” dimension (Damon et al., 2003, p: 21), although the meaning of this concept varies, with some researchers suggesting that it means the presence of pro-social, noble, or moral intentions, or behaviors designed to serve other people who are either nearby or far away (e.g., Quinn, 2012; Mariano et al., 2021). Although definitions of life purpose differ somewhat, most accommodate these three elements: commitment, goal-directedness, and personal meaningfulness (Damon et al., 2003; Bronk, 2014). Furthermore, the search and need for a life purpose are considered universal human phenomena, no matter what their cultural or specific applications (Frankl, 1959). Some scholars also suggest five roles that life purpose fulfills, including stimulating behavioral consistency, generating target motivated behaviors, triggering psychological flexibility, fostering efficient personal resources allocation, and applying higher-level cognitive processing (Yuen et al., 2015). Living a purposeful life necessitates the activation of all five portrayals to greater or lesser degrees. As such, it is reasonable to expect that purpose will be associated with these roles through student attitudes and behavior within the university experience: for example, as exhibited through consistent activity involvement and through resourcefulness.

Individuals vary in the extent to which they implement action toward their purpose. They also vary in the degree to which their purpose exhibits a prosocial focus or a focus on impacting the world beyond the self (i.e., the “other-oriented” feature discussed in some definitions). These findings reinforce the probable variation in purpose statuses and content that educators should expect to find among college students (Carver and Scheier, 2002; McKnight and Kashdan, 2009; Wang and Song, 2011). For example, individuals may commit to broad or narrow goals, prosocial or more self-oriented goals, or goals related to different fields of interest (i.e., like cleaning up the oceans or curing cancer; Biggins, 2018). Finally, one’s purpose in life appears to be influenced by age and cognitive ability, however purpose development is also determined in part by contextual factors, such as informal and informal learning experiences provided through family, culture, peers, and schooling (Damon, 2008; Moran, 2009; Pfund and Lewis, 2020). Therefore, as one highly visible learning experience, it is reasonable to expect that degree of involvement in EAs during college will be associated with students’ purpose.

Chinese perspectives have underscored the complexity of purpose, with some thinkers emphasizing purpose’s multi-level and multi-dimensional features. For example, some scholars distinguish between aspirational and more concrete approaches to purpose development. Yet, they also highlight a necessary harmony and organic unity of these two approaches (Gu, 1990; Zhang, 2006). In its broadest sense, a purpose can be expressed as a sense of yearning, akin to the idea of higher aspirations, dreams, intuitions, or hopes for the future. This notion of purpose may be closer to the broader construct of meaning that is mentioned in some of the literature (i.e., Steger et al., 2006; and see Damon et al., 2003 for a discussion), or to a *sense* of purpose (i.e., in contrast to a clearly identified purpose; see Pfund et al., 2020 for a discussion). In a more concrete or narrow sense, Chinese scholars operationalize purpose as a blueprint for the future, which should be constructed and realized through planful and systematic action in the present moment, perhaps akin to a scientific process (Chen, 2011). In this view, a truly enlightened purpose is to a large extent to chase, through concrete action in the here and now, the intuition one has of the future and the ideals one forms for it (Luo, 2000; Liu, 2001; Luo, 2021). A transcendent, beyond-self, or other-oriented dimension for purpose from a Chinese perspective can be seen in the work of some scholars. For example, these scholars designate life purpose an exceptional social consciousness and as a spiritual phenomenon that is experienced by human beings through their practice (Zhou and Yang, 2008; Wang, 2021). Others concur with popular definitions that use a type of moral or pro-social litmus test for purpose; for example, purpose is defined as making efforts not only for personal achievement but also for social progress (Jiang et al., 2016). Since purpose involves ideals in the Chinese perspective, it also embodies people’s worldview, outlook on life, and sense of values about what one should and does strive for: this is a perspective that is shared by some purpose researchers in other parts of the world (e.g., Crandall and Rasmussen, 1975; Quinn, 2012).

### The direct study of extracurricular activities and purpose

In China, extracurricular activities (EAs) are defined as all kinds of educational and teaching activities that students consciously participate in under planned organization (Hu, 2018). EAs are viewed as a far-reaching teaching channel, which is expected to help students broaden their knowledge and cultivate their hopes for the future (Dong et al., 2013); and therein lies the potential connection of EAs to purpose among Chinese college students. In China and elsewhere, there is a substantive body of research on extracurricular activities during the school years. It should be mentioned however that in contrast to the school years, college represents a relative time of freedom for students to choose the content of their extracurricular experiences as well as their level and intensity of involvement in such activities. This occurrence, along with a change in curriculum and a growth in autonomy for most students, makes the college years a unique opportunity for purpose exploration. So, it is helpful for researchers to delineate between purpose outcomes in school and college as pertains to EAs.

With some nuances, involvement in EAs has been found to be associated with positive outcomes during college. Also, according to one theory, the time a student invests in such activities is pertinent to judge such involvement (Astin, 1984/1999). However, research has yet to fully establish a connection between EA involvement and purpose during college, and especially in the context of contemporary students’ experiences in China. One reason for this, as mentioned, is the limited attention given to purpose in higher education research (Pfund et al., 2020). Consequently, few studies measure purpose directly when investigating college outcomes. We will discuss a few representative studies here however, which provide hints that influence our expectation that broad involvement in EAs should positively relate to purpose among Chinese students.

In one study, breadth of EA involvement was found to play a paramount role in the formation of students’ perceptions of employment self-efficacy, as demonstrated through the ability to conduct effective information searches for jobs (Kanar and Bouckenooghe, 2021). This study was conducted with Canadian, not Chinese, college students and did not directly assess purpose. Nonetheless, this study suggests that the more broadly students participated in EAs the more they were able to take concrete actions toward their future career paths. This may be indicative of a purposeful orientation to the future. It is also reminiscent of the view that purpose implies pairing the realization of future dreams with concrete actions, which is held by some Chinese scholars (e.g., Chen, 2011).

In a couple of other studies, reflective learning activities that involve or are guided by others were associated with purpose or related skills. Bundick (2011a) had American college students reflect upon their values, life goals and purpose through guided discussion. Students who engaged in such discussion improved in their goal directedness and life satisfaction as compared to a group that did not do the reflection. Although this study was not conducted with Chinese students, the activity described is reminiscent of what appears to go on in some of the EAs on which we gathered information at Chinese universities, as shall be described further on. In another example, Malin (2022) conducted a person-centered analysis of survey responses from over two thousand American college students. This research found that students who talked with advisors about future plans and ways to connect their strengths to something that the world needs were more likely to be assigned to a high purpose class profile. While this study was not conducted with Chinese students and does not specify EA activities, it suggests that EAs that include reflective experiences may relate to purpose.

In another study, Bundick (2011b) specifically examined positive youth development outcomes associated with a wide variety of EAs assessed twice over a 2-year period. This specified and assessed both a variety of EAs and purpose. The study mostly includes purpose as a part of an omnibus measure of positive youth development and does not directly address our questions due to its population and focus. However, this research found that creative arts activities were not associated with positive development; a finding that is pertinent to the present study because aesthetic activities are an integral part of a holistic model of college EAs. Contrary to this finding, we expect that broad and holistic EA involvement should be associated with purpose among Chinese students. Others, such as Malin (2015) have proposed that the arts can provide a rich context for purpose development, but that arts experiences may need to be uniquely tailored to individuals’ interests to be effective in this way.

### Research questions and hypotheses

Based on the foregoing, we hypothesized that students would cluster into different groups based on their patterns of extracurricular involvement in organized activities (EAs) during college. We expected that a wider variety of extracurricular activities (i.e., signifying well-roundedness) would be positively associated with purpose and thriving, which are both features of optimal development. Whether students would vary in their extracurricular involvement based on their demographic characteristics remained an open question, but we felt it was important to establish this point in order to fully understand the diversity of Chinese students’ interests and proclivities. Thus, this study addressed the following questions:

1. Can students be clustered according to their frequency of involvement in types of college organized extracurricular activities (EAs)?
2. If students can be clustered by EAs, are these students similar, or do they vary, in the following ways:

- by their purpose in life (i.e., purpose content, purpose identification, and purpose search)?
- by thriving attributes (resilience/resourcefulness and life satisfaction)?
- by the relationships exhibited between their purpose in life (content, identification, and search), and thriving attributes (resilience/resourcefulness and life satisfaction)?
- by their demographic characteristics including gender, urban or rural home town, and year in college?

## Materials and methods

### Participants and procedure

This research is part of a larger study on purpose in life among Chinese college students. All participants were recruited on their campuses during fall 2018 with the assistance of their university’s student affairs staff. Students were then invited to take an online survey by the first and third authors, who were Ph.D. candidates studying moral education and who had been trained in the data collection procedures. These researchers explained the study and online survey procedure to participants. The respondents then voluntarily completed the survey on their own time and without monitoring through an online survey program, Exam Star. All participants consented to participate on the second screen. The entire survey took a median time of 31 mins to complete. No incentives were offered in the data collection process. Participants (*N* = 332) were undergraduate students studying at 20 universities in China located in eastern, western, southern, northern and central regions of the country. They represent diverse types of colleges according to the Chinese classification system, including first-class and other national key universities as well as provincial-level universities. Three hundred and forty questionnaires were distributed, 332 of which were completed in full with no missing responses and used in the current analysis (completion rate = 97.6%). Among the participants, 148 were male (44.6%) and 184 were female (55.4%). They were 140 college first year (42.2%), 79 second year (23.8%), 64 third year (19.3%), and 49 fourth year students (14.8%). One hundred and ninety-four students came from urban areas (58.4%) and 138 came from rural areas (41.6%).

### Measures

Questionnaires derived from the Revised Youth Purpose Survey (Bundick et al., 2006) were used to assess respondents’ self-reported perceptions of distinct features of their life purpose (purpose content, purpose identification, and purpose search), thriving (resourcefulness/resiliency and life satisfaction), and frequency of involvement in college-organized extracurricular activities. The original English versions of these items were based on frequently used measures (e.g., Steger et al., 2006; see Bronk et al., 2009, p: 503). To adapt these existing tools to the Chinese college student context, questions underwent a process of examination for linguistic and cultural relevance prior to administration, informed by recommended practices in the field (e.g., Cheung et al., 2020). First, the measures were translated by two lecturers proficient in English and Mandarin who had also completed their doctorates in moral and civic education. Back-translation was then conducted by a Chinese graduate student who was completing their PhD in education in the United States. The survey was also piloted among 150 undergraduate students at two universities, and information was collected on the feasibility and interpretability of the questions. As a result of these procedures, some of the survey questions were reworded to reflect the Chinese student context. The most extensive modifications, additions, and deletions were for items assessing extracurricular involvement, because these activities in China were different in definition and scope than in the Stanford questionnaire. In a final step, the revised Chinese version of the measures were reviewed and approved by two Chinese professors who were experts in moral education. These measures are described next as they are rendered in English, with examples of adapted questions.

### Organized extracurricular activities

As described above, items from the Activity Involvement scale from the Stanford survey served as a foundation for constructing items that more precisely reflect the nature and variety of formal organized extracurricular activities at Chinese universities (see Table 1). The Stanford measure included formal or informal out-of-class time activities experienced by American young people, ordinarily classified around themes of spending time with family, volunteer service (i.e., working with children, the elderly, or those in need on one’s neighborhood), faith or spirituality (i.e., meditating, attending religious services), learning or leisure activities outside of class (i.e., attending academic clubs) or more general extracurricular engagements (e.g., sports, the arts). For the current study, several items were removed or revised. For example, items pertaining to family time were removed altogether because they did not constitute university-organized experiences. Additional items were borrowed then modified from the original. For instance, “studying/doing homework” became, “joining activities related to a class or homework.” Faith/spirituality items in the Stanford measure were recrafted to capture formal activities for cultivating Chinese students’ reflection on their personal beliefs, including new items like, “taking classes related to personal beliefs” and “joining extracurricular lectures related to personal beliefs.” A total of 24 items remained following exploratory factor analysis (discussed further on; Table 2) and constitute the Chinese Extracurricular Activities Participation (EAP) scale used for the current study. Respondents indicate how frequently they participate in each activity on a 6-point scale (1 = Never, 2 = Just once, 3 = Once a year, 4 = Once a month, 5 = Once a week, 6 = A few times a week).

**Table 1:** Factor structure of the original 27 item Extracurricular Activities Participation Scale.

Activity	Communalities	Factor loadings				
		1	2	3	4	5
Joining activities related to a class or homework	0.494	0.011	0.015	**0.686**	−0.126	0.088
Writing	0.412	0.180	0.140	**0.389**	0.091	**0.448**
Using computers or other high-tech products	0.419	−0.031	−0.087	**0.635**	−0.077	0.039
Meditating	0.467	−0.049	0.190	**0.643**	−0.029	0.121
Exercising	0.535	0.010	0.008	**0.730**	−0.004	0.040
Listening to music	0.397	−0.066	−0.038	**0.569**	0.210	0.152
Volunteering in the neighborhood	0.537	**0.668**	0.285	−0.093	0.022	0.012
Working for environmental protection	0.428	**0.597**	0.131	−0.022	0.220	0.077
Joining vocational training	0.418	**0.609**	0.094	−0.025	0.190	0.048
Volunteering for children	0.640	**0.775**	0.126	−0.040	0.110	−0.095
Participating in political activities	0.436	**0.539**	0.252	−0.158	0.234	0.053
Joining an academic club	0.492	**0.659**	0.158	0.027	0.176	0.044
Volunteering for the elderly	0.655	**0.768**	0.157	−0.086	0.180	−0.029
Meeting with professional class teachers	0.415	**0.537**	0.041	0.190	−0.157	0.252
Organizing student activities	0.495	**0.484**	0.145	−0.042	0.030	0.487
Volunteering for those in need	0.433	**0.549**	0.220	0.122	0.150	0.214
Attending extracurricular lectures related to personal beliefs	0.555	0.305	**0.618**	−0.151	−0.035	0.238
Reading books about personal beliefs	0.746	0.237	**0.820**	0.103	0.082	−0.008
Reflecting on beliefs and future aspirations	0.705	0.127	**0.802**	0.196	0.064	−0.057
Thinking about faith	0.578	0.252	**0.694**	0.048	0.155	0.079
Participating in activities related to personal beliefs	0.618	0.325	**0.605**	−0.214	0.239	0.210
Engaging in artistic creation	0.475	0.273	0.209	0.083	**0.590**	−0.046
Dancing	0.592	0.214	0.042	−0.018	**0.737**	−0.020
Attending a play/theatre/stage production	0.659	0.234	0.157	−0.189	**0.625**	0.392
Working for pay	0.299	**0.531**	0.100	0.017	0.007	0.084
Actively participating in class-related activities	0.486	0.260	0.042	0.283	−0.099	**0.572**
Listening to spiritual music	0.447	−0.133	0.026	0.203	0.098	**0.615**
Percent of common variance explained		25.52%	10.96%	6.18%	4.64%	3.94%
Total common variance explained		51.24%				

*N* = 332. Extraction method: Principal component analysis. Rotation method: Varimax with Kaiser normalization; Rotation converged in 6 iterations.

Bold values indicates significant dependence of items on relevant factor.

**Table 2:** Factor structure of the revised 24 item Extracurricular Activities Participation Scale.

Activity	Communalities	Factor loadings			
		1	2	3	4
Joining activities related to a class or homework	0.509	−0.005	0.009	**0.706**	−0.102
Writing	0.365	0.226	0.138	**0.512**	0.179
Using computers or other high-tech products	0.410	−0.021	−0.090	**0.620**	−0.131
Meditating	0.481	−0.037	0.182	**0.666**	−0.048
Exercising	0.512	−0.007	0.005	**0.714**	−0.035
Listening to music	0.372	−0.043	−0.044	**0.585**	0.161
Volunteering in the neighborhood	0.567	**0.686**	0.290	−0.111	0.004
Working for environmental protection	0.435	**0.615**	0.134	−0.023	0.196
Joining vocational training	0.404	**0.586**	0.099	−0.016	0.226
Volunteering for children	0.587	**0.743**	0.130	−0.078	0.110
Participating in political activities	0.437	**0.535**	0.250	−0.141	0.263
Joining an academic club	0.510	**0.677**	0.151	0.027	0.168
Volunteering for the elderly	0.644	**0.760**	0.165	−0.125	0.154
Meeting with professional class teachers	0.391	**0.567**	0.051	0.239	−0.100
Organizing student activities	0.356	**0.538**	0.149	0.120	0.172
Volunteering for those in need	0.449	**0.584**	0.217	0.175	0.172
Attending extracurricular lectures related to personal beliefs	0.500	0.325	**0.622**	−0.067	0.058
Reading books about personal beliefs	0.735	0.230	**0.818**	0.088	0.070
Reflecting on beliefs and future aspirations	0.686	0.094	**0.807**	0.156	0.046
Thinking about faith	0.577	0.246	**0.697**	0.058	0.164
Participating in activities related to personal beliefs	0.600	0.321	**0.607**	−0.131	0.335
Engaging in artistic creation	0.401	0.241	0.201	0.061	**0.547**
Dancing	0.560	0.175	0.032	−0.029	**0.726**
Attending a play/theatre/stage production	0.644	0.238	0.155	−0.051	**0.749**
Percent of common variance explained		27.46%	11.27%	6.76%	5.06%
Total common variance explained		50.54%			

*N* = 332. Extraction method: Principal component analysis. Rotation method: Varimax with Kaiser normalization. Rotation converged in 4 iterations. Bold values indicates significant dependence of items on relevant factor.

### Purpose features

Three features of students’ purpose in life were assessed, namely their purpose content, purpose identification, and purpose search.

#### Purpose content

To assess this feature of purpose, also called purpose category or purpose domain in some research, participants completed the Categories of Identified Purpose (CIP) scale. The measure begins with the prompt, “‘purpose refers to the MOST IMPORTANT overall goal or goals for your life,” followed by the words” The purpose of my life is to….”. Respondents then endorse a list of 17 life goals on a 7-point Likert scale (1 = strongly disagree to 7 = strongly agree; see Table 3). Items were originally derived from seminal research on categories of meaning with adolescents and young adults and have since been used in a few studies (e.g., Abramoski et al., 2018). The Chinese version used in this study modified the category item “serve God or a higher power” to read “serve my belief(s)” in order to achieve closer affinity with the non-religious terminology used for beliefs in the Chinese context. For this study, measure translation and piloting followed the procedures explained under Participants and Procedures. Analyses and Results sections also describe exploratory factor analyses conducted with this measure in the current study, resulting in 14 items (see Tables 3, 4). This measure consequently resulted in using student scores on two distinct purpose content components: *narrow self and other focused purpose* (narrow purpose) and *broad self and other focused purpose* (broad purpose).

**Table 3:** Factor structure of the original 17 item Categories of Identified Purpose scale.

The Purpose of My Life Is to...	Communalities	Factor loadings		
		1	2	3
Create something new	0.642	0.211	**0.759**	0.145
Serve my country	0.566	0.315	**0.665**	−0.154
Change the way people think	0.546	−0.192	**0.686**	0.195
Help others	0.481	0.316	**0.617**	−0.012
Make the world a better place	0.564	0.343	**0.630**	0.222
Discover new things about the world	0.490	0.308	**0.540**	0.322
Be successful	0.548	**0.689**	0.217	0.164
Have a good career	0.387	**0.617**	0.022	0.073
Fulfill my duties	0.442	**0.590**	0.302	−0.051
Do the right thing	0.554	**0.732**	0.121	−0.049
Make money	0.543	**0.617**	−0.025	0.403
Earn the respect of others	0.581	**0.717**	0.203	0.162
Support my family and friends	0.560	**0.697**	0.270	−0.036
Live life to the fullest	0.584	**0.690**	0.298	−0.136
Have fun	0.374	0.235	0.203	**0.527**
Serve my beliefs	0.635	−0.147	−0.080	**0.779**
Make things more beautiful	0.399	0.031	0.195	**0.600**
Percent of common variance explained		33.22%	10.70%	8.39%
Total common variance explained		52.33%		

*N* = 332. Extraction method: Principal component analysis. Rotation method: Varimax with Kaiser normalization (orthogonal). Rotation converged in 5 iterations. Bold values indicates significant dependence of items on relevant factor.

**Table 4:** Factor structure of the revised 14 item Categories of Identified Purpose scale.

The Purpose of My Life Is to...	Communalities	Factor loadings	
		1	2
Create something new	0.652	0.196	**0.783**
Serve my country	0.473	0.311	**0.614**
Change the way people think	0.585	−0.204	**0.737**
Help others	0.434	0.323	**0.574**
Make the world a better place	0.555	0.339	**0.663**
Discover new things about the world	0.477	0.298	**0.623**
Be successful	0.537	**0.681**	0.271
Have a good career	0.380	**0.614**	0.053
Fulfill my duties	0.430	**0.595**	0.277
Do the right thing	0.549	**0.732**	0.114
Make money	0.379	**0.610**	0.085
Earn the respect of others	0.576	**0.717**	0.248
Support my family and friends	0.553	**0.703**	0.243
Live life to the fullest	0.542	**0.690**	0.257
Percent of common variance explained		39.01%	11.85%
Total common variance explained		50.86%	

*N* = 332. Extraction method: Principal component analysis. Rotation method: Varimax with Kaiser normalization (orthogonal). Factor 1 = Narrow self and other focus; Factor 2 = Broad self and other focus. Rotation converged in 3 iterations. Bold values indicates significant dependence of items on relevant factor.

#### Purpose identification

The Purpose Identification scale incorporates 5 items (*α* = 0.716) examining the degree to which participants have already found a satisfying life purpose. It is measured by a 7-point Likert scale (1 = strongly disagree to 7 = strongly agree). These 5 items include “My life has no clear purpose” (reversely coded), “My life has a clear sense of purpose,” “I understand my life’s meaning,” “I have discovered a satisfying life purpose,” and “I have a good sense of what makes my life meaningful.” Higher ratings indicate that students are more likely to have found a satisfying purpose in life.

#### Purpose search

The Seeking Purpose scale encompasses 5 items (*α* = 0.768) testing the degree to which participants are searching for meaning or purpose in life. It is measured by a 7-point Likert scale (1 = strongly disagree to 7 = strongly agree). These items include “I am always searching for something that makes my life more meaningful,” “I am seeking a purpose or mission for my life,” “I am searching for meaning in my life,” “I am always looking to find my life’s purpose,” and “I am always looking for something that makes my life feel meaningful.” Higher ratings indicate that students are more likely to be exploring their calling in life.

### Thriving attributes

#### Resourcefulness/resiliency

The Resourcefulness/Resiliency scale (*α* = 0.766) which is part of the Thriving Assessment from the Stanford survey encompasses 4 items testing participants’ ability to adapt to life challenges. It is measured by a 7-point Likert scale (1 = strongly disagree to 7 = strongly agree). These items include “I can usually handle whatever comes my way,” “If someone challenges me, I can usually find a way to get what I want,” “I am confident that I could deal with unexpected events” and “I believe I can make it through anything, no matter what comes against me.” Higher ratings indicates that students are more likely to view themselves as adapting to challenges or unexpected events.

#### Life satisfaction

Also used in the Stanford survey’s Thriving Assessment, The Satisfaction with Life Scale (Diener et al., 1985) encompasses 5 items testing participants’ cognitive appraisal of how satisfied they are with their current life. It is measured by a 7-point Likert scale (1 = strongly disagree to 7 = strongly agree). These items include “In most ways my life is close to my ideal,” “The conditions of my life are excellent,” “I am satisfied with my life,” “So far I have gotten the important things I want in life,” “If I could live my life over, I would change almost nothing” (*α* = 0.741) Higher scores indicate that students are satisfied with their individual lives.

## Analysis

SPSS 25.0 was used for all statistical analysis. Before proceeding to answer our research questions, we explored whether the large number of items in the Categories of Identified Purpose (CIP) scale and in the Extracurricular Activities Participation (EAP) scale could be reduced. First, responses on these measures were assessed for their suitability for exploratory factor analysis. For the 17-item CIP scale, standardized deviations are all within ±2, indicating non-heteroscedasticity. For the 27-item EAP scale, standardized data processing established that all standard deviations for the respective items were within ±2, indicating non-heteroscedasticity.

Exploratory factor analysis (EFA) has been conducted before on the CIP scale with American adolescent student samples (Mariano and Savage, 2009; Abramoski et al., 2018). These studies suggest a viable underlying structure to the scale items, with the solutions yielding distinct factors dividable by self-related focus (e.g., self-oriented or beyond-the-self), or breadth (e.g., narrow versus broad; responsibility to close others or service and creativity in the larger world). We expected students’ purpose content to also meaningfully factor among our sample of Chinese college students, but also expected those themes to be populated with different items. This prediction was premised on findings from exploratory research conducted by the second and fourth authors (not reported here) and different cultural interpretations of similar items used in other scales (see Wang et al., 2022, for a discussion). As for the EAP scale, to our knowledge the extracurricular activity items have only been sorted conceptually as of this writing, making EFA pertinent in this study. Further, the original EAP scale was designed for American students, so its adaption for Chinese students is appropriate. At face value, the EAP items capture distinct types of activities and we therefore expected an EFA to also yield distinct and interpretable domains.

An EFA using a principal component (PC) method was conducted on the responses from the CIP scale and the EAP scale separately. In each case, we ran both oblique (promax) and orthogonal (varimax) rotations. However, since these analyses yielded the same interpretations for both scales, we used the orthogonal solutions. An orthogonal approach maximizes interpretability and is appropriate since the primary concern of our exploratory analysis was to identify theoretically meaningful sub-dimensions in each scale (Kim and Mueller, 1978). Factor selection for both scales adopted the method of eigenvalue greater than one. As expected, the PC analyses identified distinct and meaningful component structures for CIP and EAP scales (reported in Results), thereby allowing us to proceed to address our first research question.

For question 1, about existence of student clusters by their extracurricular activity (EA), we investigated whether assumptions were fulfilled in our data to conduct a K-means cluster analysis. For the following analyses, assumptions of normality (using QQ-plots) and homogeneity of variances (using Bartlett tests) were indeed satisfied, so we ran a K-means cluster analysis by entering EA component scores from the EFA for each student. This analysis in turn yielded interpretable clusters. For questions 2a, 2b, and 2c, we sought to understand cluster membership based on student’s purpose features and thriving attributes. After confirming that all assumptions were fulfilled to inform selection of further appropriate statistical tests, we then conducted a series of One-Way Analyses of Variance (ANOVAs) with follow-up LSD multiple comparison tests to compare the cluster groups on each of the purpose and thriving attributes (Chen, 1996). For question 2d, we compared correlation coefficients between purpose features and thriving attributes across the clusters. Finally, for question 3, frequencies and means were calculated for each cluster on selected demographic variables (gender, urban/rural/homedown, lower or upper-divison status) and Chi Square tests of independence were used to examine cluster differences by these variables. The results are reported next.

## Results

### EFA of life purpose content items

As expected, the 17 items of the Categories of Identified Purpose scale were further classified into conceptually relevant components through EFA. Three principal components initially emerged. However, the third component which included the items “having fun,” “serving God/a higher power” and “making things more beautiful” was finally discarded: These items exhibited low internal consistency (*α* = 0.485). The two retained components include two interpretable categories of life purpose content representing different foci. Frequently, a self-oriented focus and an other-oriented focus are labeled as two distinct factors in the research. However, given the emergent factor structure within a Chinese perspective of self (e.g., Wang et al., 2022) it made more sense to interpret a self-focus and beyond-self focus as present within *both* factors, yet with each factor differing in relative breadth. The decision can be explained the fact that considering Chinese perspectives, personal life purpose refers to the pursuit of individual’s future material life and spiritual life based on certain historical conditions and social relations. Social purpose refers to the common purpose of social collective and the common struggle goal that occupies the dominant position in the whole society. The relationship between personal life purpose and social purpose is the reflection of the relationship between individuals and society in the aspect of purpose. Personal life purpose is guided by social purpose. People achieve their personal life purpose within certain social contexts. At the same time, social purpose is the convergence and sublimation of personal life purpose. To sum up this idea, personal life purpose and social purpose are linked together in the Chinese perspective. They are interdependent, unified and developed together (Editorial Group, 2021). Therefore, the first component, *narrow self and other focused purpose* (called *narrow purpose* from hereon), includes items often considered more self focused, like “be successful” *as well* as items that are focused on other people or targets, such as “support my family and friends.” However, in comparison to the second component, *broad self and other focused purpose* (called *broad purpose* from hereon, *α* = 0.792) these items are relatively narrow in scope. For example, “support my family and friends” (component 1) is focused on having a positive impact on close others, whereas a corresponding other focused item in component 2, “help others” references a larger and broader category of others who could be close to *or* farther away from oneself. As was noted, oblique and orthogonal solutions yielded comparable results. Tables 3, 4 report the varimax (orthogonal) rotation. The final 14 item solution explained over 50% of the total variance (KMO = 0.85; Bartlett’s tests *p* < 0.001).

### EFA of extracurricular activities items

Also as expected, the Extracurricular Activities scale factored into clear components. The one item, “working for money,” was low in communality (<0.35) and thus removed (Hair et al., 1998). A 5 component structure next emerged with the remaining 26 items, however, one component including the items “actively participating in class activities” and “listening to spiritual music” was discarded due to lack of a common explanatory factor. A 24 item four component structure was finally retained, explaining over 50% of the total variance (KMO = 0.88; Bartlett’s Tests *p* < 0.001). The 4 components were *voluntary activiti*es (*α* = 0.859), *knowledge and skill improvement activities*, (*α* = 0.687), *belief-related activities* (*α* = 0.826) and *aesthetic activities* (*α* = 0.603; see Tables 1, 2).

### Extracurricular activity clusters

To reiterate, our first research question asked whether students could be meaningfully clustered by their extracurricular activity involvement, and a K-means cluster analysis was applied for this purpose to students’ scores on the EA components derived from an EFA. We ran solutions with K = 2, 3, 4, 5, 6, 7, 8, and 9 clusters. We then compared these solutions by analyzing *p* values from ANOVA and multiple comparison tests and by analyzing iteration tables, thus settling on the 4-cluster solution [volunteer service *F*(3, 328) = 140.80, *p* = 0.000]; knowledge and skill improvement *F*(3, 328) = 4.59, *p* = 0.004; belief-related activities *F*(3, 328) = 251.35, *p* = 0.000; aesthetic activities *F*(3, 328) = 255.02, *p* = 0.000.

Table 5 lists mean values of each cluster on the EA activities. Bundick et al. (2011b, p: 61) suggest a lack of consensus in the literature about how often one must participate in activities for them to confer benefit. We considered reports of participating about once a month (4 on our scale) to be frequent enough to indicate a student’s repeated involvement during the academic year. Thus, for interpretive purposes, means of about 4 or greater indicate frequent commitment of the individuals within a cluster overall (i.e., even with some within-cluster variation). In contrast, ratings of 3 (once a year) indicate more sporadic commitment, and ratings of 2 (just once) or 1 (never) indicate lack of commitment or no activity, respectively.

**Table 5:** Mean participation in extracurricular activities ratings by cluster.

Component	Cluster 1 Highly involved and well-rounded (N = 77)	Cluster 4 Highly involved and somewhat well-rounded (N = 88)	Cluster 2 Moderately involved (N = 77)	Cluster 3 Least involved and not well-rounded (N = 90)
Volunteer activities	4.98	3.80	3.48	2.42
Knowledge and skill improvement activities	5.49	5.72	5.49	5.40
Belief-related activities	4.90	4.38	2.59	1.97
Aesthetic activities	4.58	2.17	3.70	1.74

Before describing each cluster, it should be noted that although multiple comparison tests show statistically significant different between-cluster participation means on the knowledge and skills component, means for every cluster on this variable were still very frequent, ranging from 5.40 and 5.72. These ratings indicate participation between once and a few times a week (5 and 6, respectively, on our scale). We therefore did not consider cluster differences in the knowledge and skill improvement activities to be practically meaningful. Within each cluster, the knowledge and skill component had the highest ratings in comparison to the other components. Frequent participation on this component is not surprising given that these activities directly support academic achievement; as such, they may not inform well-rounded development and are not considered in differentiating our cluster groups. It should also be mentioned that cluster descriptions are useful in relative terms, one to another. Next, we describe each cluster. Two of the clusters (Cluster 1 and Cluster 4) have student members that are relatively more involved and well-rounded, whereas students in the other two clusters (Cluster 2 and Cluster 3) show relatively less involvement and well-roundedness.

### More involved and well-rounded clusters

Cluster 1: Highly Involved and Well-rounded (HIW). We labeled this cluster as *highly involved and well-rounded.* Mean participation ratings on the three components of volunteer activities, belief related activities and aesthetic activities were all above our threshold of 4 (once a month) and all were closer to 5 (once a week; *M* = 4.98–4.90). These means indicate a group of students who show commitment to a wide variety of types of organized extracurricular activities.

Cluster 4: Highly Involved and Somewhat Well-rounded (HISW). We labeled this cluster as *highly involved and somewhat well-rounded.* Mean participation ratings in this group were the second highest among the clusters for volunteer activities and for belief-related activities. The mean rating for volunteer activities was just slightly below the threshold of once a month (rating of 4), whereas the mean rating for belief-related activities was above it. However, the mean rating of just once for aesthetic activities (2 on our scale) was the second lowest among the clusters, clearly indicating sporadic involvement in this activity component. This cluster therefore makes up a group that may be meaningfully engaged in a variety of organized extracurriculars, with the exception of aesthetic activities.

### Less involved and less well-rounded clusters

Cluster 2: Moderately Involved (MI). We labeled this cluster as *moderately involved.* Means for volunteer activities and aesthetic activities fell mid-way between reports of once a year (3 on our scale) and our threshold of 4, once a month (i.e., 3.48 for volunteer and 3.40 for aesthetic). However, involvement in belief related activities was nearly absent by our criteria, falling about mid-way between participating just once and once a year. This group reported the second lowest mean on belief related activities in comparison with the other clusters. It seems therefore that this group could be relatively well rounded in terms of variety of their activities, yet show relatively little meaningful engagement with them, as indicated by the relatively low frequency of participation in most of the three components mentioned (i.e., volunteer, belief, and aesthetic).

Cluster 3: Least Involved and Not Well-rounded (LINW). We labeled this cluster as *least involved and not well rounded.* Participation ratings on volunteer, belief, and aesthetic activity components all fell far below the threshold of 4, once a month. The highest mean for this cluster among these components was for volunteer activities, with frequency participation rated as just above 2 (just once; *M* = 2.42). Ratings for belief and aesthetic activity components however, both fell below 2. This cluster reported the lowest mean ratings on the three components under discussion when compared to the other clusters.

### Differences in purpose and thriving by cluster

Our second question asked whether extracurricular involvement *via* cluster membership can explain students’ purpose content, purpose identification, and purpose search. As expected, purpose was positively related to more frequent and more well-rounded extracurricular involvement. The two more involved and well-rounded clusters (HIW, *M* = 5.52; HISW, *M* = 5.43) had significantly higher means on broad purpose than the less involved clusters [MI, *M* = 5.19; LINW, *M* = 5.20; *F*(3, 328) = 3.83, *p* < 0.01, *η*^2^ = 0.032], whereas no differences were found for narrow self and other purpose (HIW, *M* = 6.12; HISW, *M* = 6.22; MI, *M* = 6.18; LINW, *M* = 6.13). The more involved and well-rounded clusters (again, HIW Cluster and HISW Cluster) exhibited significantly higher purpose identification means [HIW, *M* = 5.49; HISW, *M* = 5.53; MI, *M* = 5.23; LINW, *M* = 4.93; *F*(3, 328) = 8.46, *p* = 0.000, *η*^2^ = 0.072], and higher purpose search means [HIW, *M* = 5.11; HISW, *M* = 5.00; MI, *M* = 4.94; LINW, *M* = 4.57; *F* (3, 328) = 5.68, *p* < 0.000, *η*^2^ = 0.049] than the other two clusters (MI Cluster and LINW Cluster).

We also asked whether extracurricular involvement is associated with thriving features of resourcefulness/resiliency and life satisfaction. Contrary to expectations, clusters were not significantly different in resourcefulness/resiliency (HIW, *M* = 5.01; HISW, *M* = 5.14; MI, *M* = 4.83; LINW, *M* = 4.84). However, as expected, clusters had significantly different mean life satisfaction ratings [*F*(3, 328) = 6.21, *p* = 0.000, *η*^2^ = 0.054]. Students in the highly involved and well-rounded cluster (HIW Cluster, *M* = 4.83) were significantly more satisfied with life than students in all other clusters. The next highest life satisfaction means were for MI Cluster (*M* = 4.51) and HISW Cluster (*M* = 4.50), which were not significantly different. The least involved and not well-rounded cluster (LINW Cluster, *M* = 4.17) exhibited the lowest mean ratings in life satisfaction, and its rating was significantly lower than all other clusters.

### Purpose and thriving correlations by cluster

Next, we examined whether the EA student clusters would vary in the relationships exhibited between purpose and thriving variables. Among the full sample, all purpose features were positively and significantly correlated with the two thriving measures, with the exception of narrow purpose and life satisfaction. By Cohen’s (1988) standard, the statistically significant correlations were small to moderate, with the strongest associations exhibited between purpose identification and resilience/resourcefulness (*r* = 0.462), and purpose identification and life satisfaction (*r* = 0.418; see Table 6).

**Table 6:** Pearson correlations among purpose and thriving variables for the full sample.

Outcome measure	1	2	3	4	5	6
1. Purpose content—Broad self and other focus						
2. Purpose content—Narrow self and other focus	0.526^**^					
3. Purpose identification	0.363^**^	0.316^**^	-			
4. Purpose search	0.262^**^	0.208^**^	0.200^**^	-		
5. Resilience/resourcefulness	**0.326** ^**^	**0.249** ^**^	**0.462** ^**^	**0.168** ^**^	-	
Life satisfaction	**. 172** ^**^	0.064	**0.418** ^**^	**0.156** ^**^	0.415^**^	

** *p < 0.01 (2-tailed).*

Statistically significant correlations between purpose and thriving variables are in bold.

However, Table 7 shows within-cluster Pearson correlations, showing that some associations between purpose and thriving found in the full sample did not hold for all clusters. Broad purpose content and resilience/resourcefulness were moderately correlated in the full sample (*r* = 0.326, *p* < 0.001), but not in MI Cluster. A small but significant positive correlation between broad purpose content and life satisfaction in the full sample (*r* = 0.172, *p* < 0.001) appears to be explained solely by the moderate correlation between these two variables in the HISW Cluster (*r* = 0.370, *p* < 0.05). Narrow purpose content and resilience/resourcefulness, which correlated in the full sample (*r* = 0.249, *p* < 0.001), was only significantly correlated in the HIW Cluster (*r* = 0.413, *p* < 0.001) and the LINW Cluster (*r* = 0.213, *p* < 0.05). Purpose search was significantly correlated with resilience/resourcefulness only in the HIW (*r* = 0.231, *p* < 0.001) and the HISW Cluster (*r* = 0.315, *p* < 0.001; full sample *r* = 0.168, *p* < 0.001).

**Table 7:** Pearson correlations among purpose and thriving variables by cluster.

Outcome measure	Cluster 1 Highly involved and well-rounded (N = 77)	Cluster 4 Highly involved and somewhat well-rounded (N = 88)	Cluster 2 Moderately involved (N = 77)	Cluster 3 Least involved and not well-rounded (N = 90)
**1.Purpose content—broad self and other focus**				
a. x Purpose—Narrow self and other focus	0.638^**^	0.342^**^	0.648^**^	0.481^**^
b. x Purpose identification	0.552^**^	0.376^**^	0.188	0.270^*^
c. x Purpose search	0.351^**^	0.253^*^	0.189	0.176
d. x Resilience/resourcefulness	0.443^**^	0.385^**^	0.205	0.225^*^
e. x Life satisfaction	0.192	0.370^*^	0.123	−0.068
**2.Purpose content—narrow self and other focus**				
a. x Purpose identification	0.425^**^	0.257^**^	0.284^*^	0.322^**^
b. x Purpose search	0.174	0.141	0.252^*^	0.276^**^
c. x Resilience/resourcefulness	0.413^**^	0.189	0.176	0.213^*^
d. x Life satisfaction	0.215	0.196	0.021	−0.039
**3.Purpose identification**				
a. x Purpose search	0.421^**^	0.160	0.179	−0.082
b. x Resilience/resourcefulness	0.578^**^	0.437^**^	0.510^**^	0.309^**^
c. x Life satisfaction	0.290^*^	0.413^**^	0.453^**^	0.396^**^
**4.Purpose search**				
a. x Resilience/resourcefulness	0.231^**^	0.315^**^	0.206	−0.106
d. x Life satisfaction	0.154	0.196	0.141	−0.015
**5.Resilience/resourcefulness**				
a. x Life satisfaction	0.557^**^	0.453^**^	0.438^**^	0.230^*^

** *p* < 0.01 (2-tailed);

* *p* < 0.05 (2-tailed).

Furthermore, bivariate relationships between the purpose variables also varied between the full sample and the clusters. The correlation between broad purpose content and purpose identification was moderate in the full sample (*r* = 0.363, *p* < 0.001), but was not significant in MI Cluster (*r* = 0.188, *p* > 0.05). Narrow purpose content and purpose search was only correlated in MI Cluster (*r* = 0.252, *p* < 0.05) and LINW Cluster (*r* = 0.276, *p* < 0.01) but not in HIW Cluster and HISW Cluster (*r* = 0.141, *p* > 0.05); in contrast, the opposite pattern was observed for broad purpose content and purpose search, which were correlated in HIW Cluster (*r* = 0.351, *p* < 0.01) and HISW Cluster (*r* = 0.253, *p* < 0.05) but not in the other two clusters. The meaning of the patterns observed in the correlation matrices are considered in the Discussion.

### Demographic characteristics by cluster

To answer our final question, descriptive statistics for demographic characteristics were listed by cluster. Chi-square tests of independence found that clusters were significantly different by gender (**HIW**, *N*_Female_ = 37, 48.05%; **HISW**, *N*_Female_ = 47, 53.41%; **MI**, *N*_Female_ = 53, 68.83%; **LINW**, *N*_Female_ = 47, 52.22%; *χ^2^* = 7.814, *p* < 0.050, Cramers *V* = 0.05), hometown origins (urban or rural) (**HIW**, *N*_Urban_ = 49, 63.64%; **HISW**, *N*_Urban_ = 40, 45.45%; **MI**, *N*_Urban_ = 55, 71.43%; **LINW**, *N*_Urban_ = 50, 55.56%; *χ^2^* = 12.622, *p* < 0.006, Cramers *V* = 0.006), and year in college (**HIW**, *N*_First_ = 43, 55.84%; *N*_Second_ = 16, 20.78%; *N*_Third_ = 14, 18.18%; *N*_Fourth_ = 4, 5.19%; **HISW**, *N*_First_ = 28, 31.82%; *N*_Second_ = 30, 34.09%; *N*_Third_ = 17, 19.32%; *N*_Fourth_ = 13, 14.77%; **MI**, *N*_First_ = 39, 50.65%; *N*_Second_ = 18, 23.38%; *N*_Third_ = 16, 20.78%; *N*_Fourth_ = 4, 5.19%; **LINW**, *N*_First_ = 45, 50%; *N*_Second_ = 20, 22.22%; *N*_Third_ = 25, 27.78%; *N*_Fourth_ = 0, 0%, *χ^2^* = 27.944, *p* < 0.001, Cramers *V* = 0.001). Standardized residuals showed that the MI Cluster reported less males than expected than other clusters, that the HIW Cluster and the MI Cluster had higher proportions of students from urban hometowns than the other clusters, and that the HISW Cluster had fewer first-year students and more fourth-year students than expected.

## Discussion

We examined whether broad and frequent engagement in organized extracurricular activities (EAs) is associated with Chinese college students’ optimal outcomes of purpose in life, resilience/resourcefulness, and life satisfaction. In the following, we summarize the main findings and their contribution to the research area, identify implications for practice and future research, and discuss limitations.

### Assessing a variety of EAs captures student diversity beyond academic pursuits

A contribution of this study was that it includes the wide variety of types of organized extracurricular activities in Chinese universities (Bartkus et al., 2012). As such, this strategy revealed a diversity of student experience. Per our expectations, we found that the demographically diverse group of students that we surveyed from across China were also diverse in their EA involvement, as they could be clearly clustered into distinct groups. The clustering of students in this way reveals the psychological diversity of students in Chinese colleges, as measured by what students are interested in spending time doing in concert with their academic pursuits.

One interesting finding is that student clusters differed in frequency of EAs that were not directly related to academic achievement. This suggests that measures of academic-related EAs may not be useful in differentiating such students, and that a wider variety of extracurricular experiences – such as activities in which students spend time cultivating and living out their beliefs and values, community service, or aesthetic experiences – can contribute to a more robust understanding of factors that affect well-being. The findings also suggest that Chinese college students are still highly oriented toward their studies even outside of class time, and access to a wide variety of EAs is probably not distracting from this focus.

### Well-roundedness aligns with purpose and life satisfaction

We had expected that more frequent and well-rounded EA involvement would be associated with purpose and thriving. This hypothesis was supported in several ways. In regard to purpose, a clear pattern emerged around purpose content, identification, and search. First, it appears that broad purpose content (i.e., broad promotion of self and others) is linked to broad EA engagement. This was shown by the fact that the Highly Involved and Well-rounded (HIW) and Highly Involved and Somewhat Well-rounded (HISW) clusters endorsed broad purpose content significantly more than other two, less well-rounded, clusters. Other research has been occupied with the idea of other-oriented purpose or “beyond-the-self” purpose too (Liu, 2001; Wu, 2011; Luo, 2021), finding that life goals concentrated on both self *and* others are associated with positive outcomes. Our results may therefore contribute to a greater understanding of the beyond-the-self purpose construct in Chinese college students by linking beyond-the-selfness to consistency (i.e., frequency) and breadth of activity engagement. Notably, our cultural alignment and then exploratory factor analysis of items of the Categories of Identified Purpose scale also contribute to a better understanding of the beyond-the-self purpose factor from a Chinese perspective, since our two factors meaningfully combined goals for self and other, distinguished only by breadth.

Second, a pattern in support of our main hypothesis emerged around purpose identification and purpose search. Purpose identification signifies that one has discovered a purpose, and this aspect of purpose is linked to positive outcomes. In contrast, purpose search suggests one is exerting effort to find a purpose; but like purpose identification, purpose search also infers healthy exploration of one’s life path during emerging adulthood (Bronk et al., 2009; Bronk and Finch, 2010). The ability to identify a purpose for one’s life among the students positively correlated with the frequency and scope (i.e., well-roundedness) of their extracurricular involvement. This was shown by the fact that students in the more involved and well-rounded clusters (HIW Cluster and HISW Cluster) reported significantly higher purpose identification scores than the less involved clusters (MI Cluster and LINW Cluster), and that another significant decrease was apparent between the MI Cluster and LINW Cluster (i.e., with the LINW Cluster classified as the least involved). The similar pattern was exhibited for purpose search, with the least involved cluster (LINW Cluster) reporting significantly less purpose search than the other three clusters.

Another point that merits consideration is that different clusters exhibited different relationships between purpose identification and purpose search. These two aspects of purpose are found to be positively correlated among American and Chinese emerging adults (Damon et al., 2003; Bronk, 2014; Jiang et al., 2016), and it is thought that their positive relationship may be normative – and even optimal – during this time of life. In our sample however, identification and search were positively correlated only among the most involved and well-rounded students (HIW Cluster); thereby lending further support for the assertion that more well-rounded students will exhibit more optimal purpose outcomes.

This last finding about cluster differences in how purpose identification and search relate implies other important areas for investigation to foster positive student development. For instance, it could imply that those who explore a wide range of learning experiences may also be those who are searching more, and this in turn might imply that such students are more likely to adopt a view of purpose development that is dynamic rather than static. A dynamic view is consistent with what we know about the course of purpose development across the lifespan and during the period of youth and the multiple ways that purpose may grow (Malin et al., 2014; Pfund and Lewis, 2020). Promoting an understanding that one’s purpose is dynamic could also help young people sidestep pressures experienced when tasked with “finding” their purpose (e.g., Pfund et al., 2020, p: 98). Thus, we also wonder whether the patterns seen here indicate a set of broad versus narrow, and dynamic versus static, worldviews that tend to be adopted by well-rounded students, and which conceivably correlate with quality of life. This proposition was not assessed here but could be in future studies. More insights on this theme could further inform universities about the usefulness of organized EAs for supporting student growth and well-being in changing times.

Finally, in respect to thriving, the results support the hypothesis that greater EA involvement is associated with greater life satisfaction. The most well-rounded cluster (HIW Cluster) exhibited the highest life satisfaction of all the groups, and the least involved and well-rounded cluster (LINW Cluster) had the lowest life satisfaction.

### Unclear patterns may be addressed through other methods

Some of the other patterns in the findings are less straightforward to explain than those that clearly support our hypotheses. While the general patterns of EA by purpose and EA by life satisfaction were apparent across clusters, some of the finer between-cluster differences are harder to decipher. For example, in respect to life satisfaction, multiple comparison tests did set the most and least involved and well-rounded clusters (HIW Cluster and LINW Cluster respectively) apart from the others (i.e., the HIW Cluster reported the highest life satisfaction and the LINW Cluster reported the lowest). However, the MI Cluster and the HISW Cluster were not significantly different in life satisfaction. According to our lens, the pairing of these two is hard to explain. As such, patterns like this merit attention in future studies that use person-centered analyses, as we do. Maybe using other person-centered methods like latent class analysis could shed light on this matter since the grouping algorithm in that method allows for uncertainty in class membership using probabilities. Furthermore, the less straightforward patterns that appeared suggest that the findings for EA and purpose are more robust than are those for EA and life satisfaction.

Also contrary to expectations, our second thriving attribute (resilience/resourcefulness) was not related to EA *via* cluster membership. One explanation is that our brief measure of this construct combines two different ideas but captures both only partially. A more robust and extensive measure of each construct may provide further explanation on the questions in this paper. A second explanation is that EA involvement may be only indirectly related to resilience/resourcefulness, such as through purposefulness. All purpose features correlated with resilience/resourcefulness in the full sample. Therefore, a research design that identifies within-group mediation effects between EA and resilience/resourcefulness could tease out finer nuances than were found in this study.

### Limitations

We have now mentioned a number of points that limit the conclusions of our findings, including measurement of resilience/resourcefulness, and use of other person-centered methods. Clearly, our findings are also correlational in nature, warranting longitudinal designs that could specify the direction of the associations discovered here. Cluster differences by gender, hometown origin, and year in college were merely described in this study, but could be more fully examined, if not only to understand the diversity of college students in contemporary China. We have argued that the purpose of knowing about students’ diversity is to help universities address their students’ interests and proclivities through the extracurricular activities they provide for them. To this end, future studies could also gauge the quality of students’ involvement in EAs so as to improve the quality of those EA experiences for their potential effects on purpose and thriving. We assessed frequency of EA involvement in this study because it captures students’ time investment. This approach is supported in the literature, but knowing quality of students’ involvement may more fully capture their true engagement, which is turn may more fully explain students’ purposes and thriving (Astin, 1984/1999). Furthermore, although our findings support the idea that purpose and thriving are related to broad EA involvement, they do not address the question of how this is the case, except through frequency of activities. Therefore, researchers interested in our questions could borrow methods from studies that address the how of student involvement (e.g., Tinto, 1997).

Future research should consider other issues in measurement as well. For example, we propose using other assessments to gauge student happiness as a potential positive indicator of EA effects. Life satisfaction is commonly used, but some researchers point to the limitations of this construct as a measure of real happiness (e.g., Ryff, 2014). Instead, researchers might explore how students’ meaningful and broad engagement in activities support their deeper sense of joy (Johnson, 2020). This point could also extend to the study of aesthetic activities. We were interested in knowing how a holistic model of EA engagement related to students’ purpose and thriving, but other analyses could take a closer look at the role of specific EAs in these relationships among Chinese students, especially aesthetic involvement. Only the most well-rounded of the student clusters in this study exhibited frequent involvement in aesthetic EAs by our standard (i.e., a mean rating of 4, or at least once a month), and this cluster also reported significantly higher life satisfaction than the other groups. Involvement in aesthetic involvement also appears to be the main EA differentiating between the two most involved well-rounded clusters. While the pattern of findings does not suggest that aesthetic EAs are associated with greater purpose, they do hint that further investigation is needed, as echoed in previous findings about arts participation and youth thriving, even as related to purpose (i.e., Bundick, 2011b; Malin, 2015). It could be for instance that positive effects of aesthetic activities are not to be found in frequency or listing of the activities themselves, but rather in the engagement and psychological benefits that accrue when one’s aesthetic appreciation abilities are cultivated, and then applied to purposeful pursuits toward good ends (Kaufman, 2018).

## Conclusion

In China, students can choose from a variety of deep and broad learning experiences as institutions of higher education strive to cultivate all-round and whole-student growth, while also attending to the varied interests of a widening diversity of students entering their programs, and with an eye to supporting students’ thriving. From the current study, it is clear that organized extracurricular activities are consonant with this goal and an important variable to consider for promoting positive student outcomes during college. Even more specifically, how EAs support youths’ life purpose is a worthy area of study during college since purpose is associated with many positive outcomes, and college is an opportune time for students to explore their life paths. Consequently, it seems that policy makers, researchers, and educators will find themselves going in the right direction if they continue to consider how learning experiences can support students in their purpose development, in China, and everywhere.

## Data availability statement

The datasets for this study can be found in the Center for Ideological and Political Education, further inquiries can be directed to the corresponding author. Requests to access these datasets should be directed to FJ, jiangf890@nenu.edu.cn.

## Ethics statement

The studies involving human participants were reviewed and approved by the University Committee on Activities Involving Human Subjects, Northeast Normal University. The participants provided their written informed consent to participate in this study.

## Author contributions

All authors listed have made a substantial, direct and intellectual contribution to the work, and approved it for publication.

## Funding

This project was supported by a grant to Fei Jiang at Northeast Normal University from the National Social Science Foundation of China (19BKS161).

## Conflict of interest

The authors declare that the research was conducted in the absence of any commercial or financial relationships that could be construed as a potential conflict of interest.

## Publisher’s note

All claims expressed in this article are solely those of the authors and do not necessarily represent those of their affiliated organizations, or those of the publisher, the editors and the reviewers. Any product that may be evaluated in this article, or claim that may be made by its manufacturer, is not guaranteed or endorsed by the publisher.

## References

[ref1] Abramoski K. Pierce J. Hauck C. Stoddard S. (2018). Variations of adolescent purpose in life and their association with lifetime substance abuse. J. Sch. Nurs. 34, 114–120. doi: 10.1177/1059840517696964, PMID: 29512434

[ref2] Astin A. W. (1984/1999). Student involvement: a developmental theory for higher education. J. Coll. Stud. Dev. 40, 518–529.

[ref5] Bartkus K. R. Nemelka B. Nemelka M. Gardner P. (2012). Clarifying the meaning of extracurricular activity: a literature review of definitions. Am. J. Bus. Educ. 5, 693–704. doi: 10.19030/ajbe.v5i6.7391

[ref8] Biggins Y. (2018). Purpose in adolescence: A review of the literature and an intervention plan. (unpublished masters thesis). Available at: https//repository.upenn.edu/map_cpastone/149

[ref9] Bronk K. C. (2014). Purpose in life: A critical component of optimal youth development. Berlin: Springer.

[ref10] Bronk K. C. Finch R. H. (2010). Adolescent characteristics by type of long-term aim in life. Appl. Dev. Sci. 14, 35–44. doi: 10.1080/10888690903510331

[ref11] Bronk K. C. Hill P. L. Lapsley D. K. Talib T. L. Finch H. (2009). Purpose, hope, and life satisfaction in three age groups. J. Posit. Psychol. 4, 500–510. doi: 10.1080/17439760903271439

[ref12] Bundick M. J. (2011a). The benefits of reflecting on and discussing purpose in life in emerging adulthood. New Dir. Youth Dev. 2011, 89–103. doi: 10.10.1002/yd/430 22275281

[ref13] Bundick M. J. (2011b). Extracurricular activities, positive youth development, and the role of meaningfulness of engagement. J. Posit. Psychol. 6, 57–74. doi: 10.1080/17439760.2010.536775

[ref14] Bundick M. Andrews M. Jones A. Mariano J. M. Bronk K. C. Damon W. (2006). Revised youth purpose survey. [unpublished instrument]. Stanford Center on Adolescence.

[ref15] Carver C. S. Scheier M. F. (2002). Control processes and self-organization as complementary principles underlying behavior. Personal. Soc. Psychol. Rev. 6, 304–315. doi: 10.1207/S15327957PSPR0604_05

[ref16] Chen G. S. (1996). Extracurricular activities. Shanghai Research on Education. 1, 15–16.

[ref17] Chen L. S. (2011). Comparative ideological and political education. Beijing: China Renmin University Press.

[ref18] Cheung H. Mazerolle L. Possingham H. Tam K.-P. Biggs D. (2020). A methodological guide for translating study instruments in cross-cultural research: adapting the ‘connectedness to nature’ scale into Chinese. Methods Ecol. Evol. 11, 1379–1387. doi: 10.1111/2041-210X.13465

[ref19] Cohen J. (1988). Statistical power analysis for the behavioral sciences (2nd ed). New York: Lawrence Erlbaum Associates.

[ref20] Crandall J. E. Rasmussen R. D. (1975). Purpose in life as related to specific values. J. Clin. Psychol. 31, 483–485. doi: 10.1002/1097-4679(197507)31:3<483::AID-JCLP2270310326>3.0.CO;2-C

[ref21] Damon W. (2008). The path to purpose: How young people find their calling in life. New York, NY: Free Press.

[ref22] Damon W. Menon J. Bronk K. C. (2003). The development of purpose during adolescence. Appl. Dev. Sci. 7, 119–128. doi: 10.1207/S1532480XADS0703_2

[ref23] Diener E. Emmons R. A. Larsen R. J. Griffin S. (1985). The satisfaction with life scale. J. Pers. Assess. 49, 71–75. doi: 10.1207/s15327752jpa4901_13 16367493

[ref24] Dong L. Luo M. M. Qian X. (2013). Construction of a problem-oriented extra-curricular activity teaching system for ideological and political theory courses in colleges and universities. Theory Practice of Educ. 6, 58–59.

[ref25] Editorial Group. (2021). Ideological morality and rule of law. China: Higher Education Press.

[ref26] Frankl V. E. (1959). Man’s search for meaning: An introduction to logotherapy. Boston: Beacon.

[ref27] Gu M. Y., (1990). Dictionary of education. Shanghai: Shanghai Education Press.

[ref28] Hair J. F. Tatham R. L. Anderson R. E. Black W. (1998). Multivariate data analysis (5th ed.). Englewood Cliffs, NJ: Prentice-Hall.

[ref30] Hu W. S. (2018). An empirical study on the quality of university students’ learning activities in Beijing. Soc. Sci. Beijing 6, 24–37. doi: 10.13262/j.bjsshkxy.bjshkx.180603

[ref31] Jiang F. Lin S. Mariano J. M. (2016). The influence of Chinese college teachers’ competence for purpose support on students’ purpose development. J. Educ. Teach. 42, 565–581. doi: 10.1080/02607476.2016.1226555

[ref0001] Johnson M. K. (2020). Joy: a review of the literature and suggestions for future directions. J. Posit. Psychol. 15, 5–24. doi: 10.1080/17439760.2019.1685581

[ref32] Kanar A. Bouckenooghe D. (2021). The role of extracurricular activities in shaping university students’ employment self-efficacy perceptions. Career Dev. Int. 26, 158–173. doi: 10.1108/CDI-02-2020-0036

[ref33] Kaufman J. C. (2018). Finding meaning with creativity in the past, present, and future. Perspect. Psychol. Sci. 13, 734–749. doi: 10.1177/1745691618771981, PMID: 30227083

[ref34] Kim J. O. Mueller C. W. (1978). Factor analysis: Statistical methods and practical issues. Thousand Oaks: Sage.

[ref35] Lan X. X. Zhang A. M. (2021). On the three logics of the fundamental task of moral education and cultivate people. China Higher Educ. 24, 31–33.

[ref36] Liu S. (2001). Comments on the personality value purpose of “junzi” in Confucianism. J. Ningxia Communist Party Institute 3, 54–55.

[ref37] Luo G. J. (2000). Ideal faith and construction of “three views”. Beijing: Press of Party School of the Central Committee of CPC.

[ref38] Luo Y. T. (2021). Ideals and convictions are the core of the cohesion of the CPC. Stud. Ideological Educ. 4, 4–11. doi: 10.16075/j.cnki.cn31-1220/g4.2021.04.001

[ref39] Malin H. (2015). Arts participation as a context for youth purpose. Stud. Art Educ.: J. Issues Res. 56, 268–280. doi: 10.1080/00393541.2015.11518968

[ref40] Malin H. (2022). Engaging purpose in college: a person-centered approach to studying purpose in relation to college experiences. Appl. Dev. Sci. 1–16. doi: 10.1080/10888691.2022.2033120

[ref41] Malin H. Reilly T. S. Quinn B. Moran S. (2014). Adolescent purpose development: exploring empathy, discovering roles, shifting priorities, and creating pathways. J. Res. Adolesc. 24, 186–199. doi: 10.1111/jora.12051

[ref42] Mariano J. M. Damiani T. Boyer M. (2021). Self- and other-reported virtues of young purpose exemplars. Youth Soc. 53, 466–485. doi: 10.1177/0044118X19859022

[ref43] Mariano J. M. Savage J. (2009). Exploring the language of youth purpose: references to positive states and coping styles by adolescents with different kinds of purpose. J. Res. Character Educ. 7, 1–24.

[ref44] McKnight P. E. Kashdan T. B. (2009). Purpose in life as a system that creates and sustains health and well-being: an integrative, testable theory. Rev. Gen. Psychol. 13, 242–251. doi: 10.1037/a0017152

[ref45] Ministry of Education of the People’s Republic of China. (2010). Outline of the national medium- and long-term education reform and development plan (2010–2020). Available at: http://www.moe.gov.cn/srcsite/A01/s7048/201007/t20100729_171904.html

[ref46] Moran S. (2009). Purpose: giftedness in intrapersonal intelligence. High Abil. Stud. 20, 143–159. doi: 10.1080/13598130903358501

[ref48] Pfund G. N. Bono T. J. Hill P. L. (2020). A higher goal during higher education: the power of purpose in life during university. Translational Issues in Psychological Sci. 6, 97–106. doi: 10.1037/tps0000231

[ref49] Pfund G. N. Lewis N. A. (2020). “Aging with purpose: developmental changes and benefits of purpose in life throughout the lifespan,” in Personality and healthy aging in adulthood. International perspectives on aging. Vol. 26. eds. Hill P. Allemand M. (Berlin: Springer), 27–42.

[ref50] Quinn B. (2012). Other-oriented purpose: the potential roles of beliefs about the world and other people. Youth Soc. 46, 779–800. doi: 10.1177/0044118X12452435

[ref51] Reilly T. S. Mariano J. M. (2021). Fostering mature purpose beyond the classroom: considering family and other institutions in mature purpose development. Estudos de Psicologia (Campinas) 38:e210115. doi: 10.1590/1982-027520213

[ref52] Ryff C. D. (2014). Psychological well-being revisited: advances in the science and practice of Eudaimonia. Psychother. Psychosom. 83, 10–28. doi: 10.1159/000353263, PMID: 24281296 PMC4241300

[ref53] Steger M. F. Frazier P. Oishi S. Kaler M. (2006). The meaning in life questionnaire: assessing the presences of a search for meaning in life. J. Couns. Psychol. 53, 80–93. doi: 10.1037/0022-0167.53.1.80

[ref54] Tinto V. (1997). Classrooms as communities: exploring the educational character of student persistence. J. High. Educ. 68, 599–623. doi: 10.1080/00221546.1997.11779003

[ref55] Wang B. X. (2021). The task of the times and practical logic of college students’ firm ideal and belief. J. Northeast Normal University (Social Science) 5, 145–150. doi: 10.16164/j.cnki.22-1062/c.2021.05.019

[ref56] Wang Y. Song Y. W. (2011). Problems and countermeasures of ideal and faith education of college students under the new situation. Leading J. Ideological & Theoretical Educ. 4, 57–60.

[ref57] Wang T. You X. Q. Huang X. T. (2022). Chinese life purpose orientation questionnaire: assessing purpose orientations among Chinese college students. Appl. Dev. Sci. 26, 362–374. doi: 10.1080/10888691.2020.1815535

[ref58] Wong M. (2020). Changes in general education curriculum in Chinese universities. Available at: http://www.china.org.cn/opinion/2020-07/12/content_76258881.htm

[ref01] Wu Q. T. (2011). Properly Understand the Scientific Meaning of Ideal Beliefs. Teaching and Research 4, 5–9.

[ref59] Xinhua News Agency. (2020). Xi Jinping presided over the 19th meeting of the central Committee for Comprehensively Deepening Reform. Available at: http://www.xinhuanet.com/politics/leaders/2021-05/21/c_1127476498.htm

[ref61] Yuen M. Lee Q. A. Y. Kam J. Lau P. S. Y. (2015). Purpose in life: a brief review of the literature and its implications for school guidance programmes. J. Psychol. Couns. Sch. 27, 55–69. doi: 10.1017/jgc.2015.18

[ref62] Zhang Y. C. (2006). Modern ideological and political education. Beijing: People’s Publishing House.

[ref63] Zhou J. Yang M. R., (2008). An introduction to the outlook on life and values of life. Tinajin: Tianjin Science and Technology Press.

